# Characterization of Peanut Protein Hydrolysate and Structural Identification of Umami-Enhancing Peptides

**DOI:** 10.3390/molecules27092853

**Published:** 2022-04-30

**Authors:** Lixia Zhang, Xiaojing Sun, Xin Lu, Songli Wei, Qiang Sun, Lu Jin, Guohui Song, Jing You, Fei Li

**Affiliations:** 1 Research Center for Agricultural and Sideline Products Processing, Henan Academy of Agricultural Sciences, Zhengzhou 450002, China; lxzhang2003@163.com (L.Z.); 18300697186@163.com (X.S.); hellwolf2007@gmail.com (X.L.); weisongli1991@163.com (S.W.); lubyjin@foxmail.com (L.J.); sigehe@126.com (G.S.); jingyou199303@163.com (J.Y.); 2 Puyang Xunda Grain and Oil Co., Ltd., Puyang 457177, China; nanjingfei2014@163.com

**Keywords:** peanut protein hydrolysate, umami-enhancing peptide, characterization, isolation and purification, amino acid sequence

## Abstract

Umami peptides are naturally found in various foods and have been proven to be essential components contributing to food taste. Defatted peanut powder hydrolysate produced by a multiprotease (Flavorzyme, Alcalase, and Protamex) was found to elicit an umami taste and umami-enhancing effect. The taste profiles, hydrolysis efficiency, amino acids, molecular weight distribution, Fourier transform infrared spectroscopy (FT-IR), and separation fractions obtained by ultrafiltration were evaluated. The results showed that peanut protein was extensively hydrolyzed to give mainly (up to 96.84%) free amino acids and peptides with low molecular weights (<1000 Da). Furthermore, β-sheets were the major secondary structure. Fractions of 1–3000 Da and <1000 Da prominently contributed to the umami taste and umami enhancement. To obtain umami-enhancing peptides, these two fractions were further purified by gel filtration chromatography, followed by sensory evaluation. These peptides were identified as ADSYRLP, DPLKY, EAFRVL, EFHNR, and SDLYVR by ultra-performance liquid chromatography (UPLC), and had estimated thresholds of 0.107, 0.164, 0.134, 0.148, and 0.132 mmol/L, respectively. According to the results of this work, defatted peanut powder hydrolysate had an umami taste and umami-enhancing effect, and is a potential excellent umami peptide precursor material for the food industry.

## 1. Introduction

In recent years, enzymatic hydrolysis technology has been widely used in the food industry. Proteolysis, which can generate numerous peptides and free amino acids via peptide bond cleavage, is an effective method for improving the value and functional properties of proteins [1]. In addition to the biological functions, the taste properties of low-molecular-weight polypeptides have been identified in various foods. Taste peptides are oligopeptides with molecular weights lower than 3000 Da that have a special effect on taste or make a partial contribution to food flavor. Umami taste plays a crucial role in enhancing favorable flavors and pleasant tastes [2]. Therefore, umami peptides have received growing attention [3]. To date, these have mostly been derived from the enzymolysis of animal protein, such as fish [4], bovine bone [5], and clam [6]. The enhancement of umami taste by peptides has also been studied. Some researchers have recognized that many peptides have no or slight umami tastes but can significantly increase the umami intensity when added to other umami ingredients. Oh et al. [7] found that adding peptides to seafood soup or an aqueous solution with umami taste enhanced the umami intensity. Furthermore, Xu et al. [8] extracted two umami-enhancing peptides that were able to enhance the umami taste of monosodium glutamate (MSG) from *Volvariella volvacea*.

Peanut (*Arachis hypogaea* L.) is among the major oilseed crops globally used as a food, and is widely applied in the food industry owing to its multiple nutrients. Defatted peanut meal is the main byproduct of peanut oil production and is an excellent umami peptide precursor with a large amount of acidic amino acids (Arg and Glu), which have been proven to be the main compounds resulting in the umami taste [9]. Research on the taste of hydrolysates from defatted peanut flour (DPF) protein has been reported by Govindaraju and Srinivas [10]. Su et al. [11,12] discussed the hydrolysis characteristics of DPF, and obtained two novel umami peptides and umami-enhancing peptides, namely, an octapeptide and undecapeptide, by hydrolysis of a protease extract from the fermentation of *Aspergillus oryzae*.

In the present study, DPF was hydrolyzed by a multiprotease (Flavorzyme, Alcalase, and Protamex). The hydrolysis efficiency and taste traits of the DPF hydrolysates (DPHs) were evaluated. Amino acids (AAs) and free amino acids (FAAs), molecular weight (MW) distribution, and Fourier transform infrared spectroscopy (FT-IR) measurements were also conducted to track protein structure changes caused by hydrolysis. Furthermore, DPH was separated and purified by ultrafiltration and gel filtration chromatography (GFC). Ultra-performance liquid chromatography (UPLC) was conducted to determine the amino acid sequences of the umami-taste fractions. These identified peptides were further subjected to synthesis and sensory evaluation to analyze the umami-enhancing effects, facilitating the discovery of more novel umami-enhancing peptides from DPF.

## 2. Results and Discussion

### 2.1. Enzymatic Hydrolysis Characteristics of DPF

**Figure 1:**
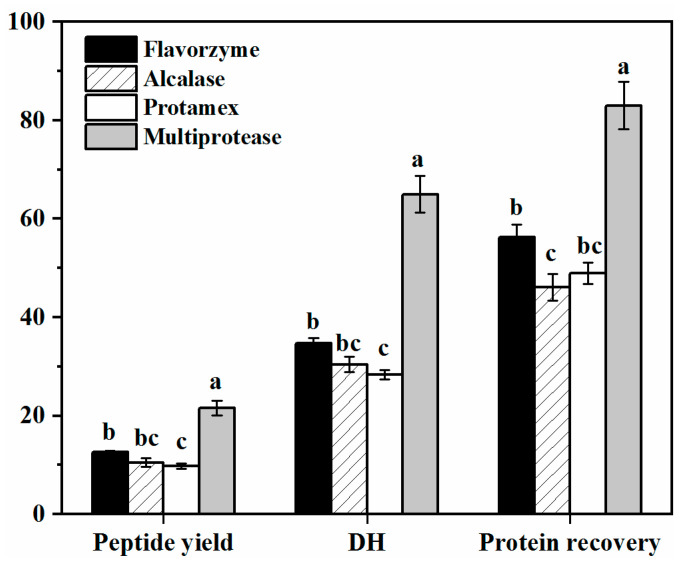
Taste characteristics of DPH. Different letters denote significant differences (*p* < 0.05).

The protein recovery reflected the utilization rate of DPF protein while the degree of hydrolysis (DH) and peptide yield reflected the degree of protein hydrolysis, which were used to characterize the hydrolysis efficiency of proteases. The protein recovery, DH, and peptide yield obtained from protease hydrolysis of DPF are shown in Figure 1.

From Figure 1, the positive effect of adding multiprotease preparations was significantly greater than that of adding a single enzyme (*p* < 0.05). So, the multiprotease was selected in this study. Under the action of a highly efficient multiprotease, a large number of intermediate peptides were produced from the peanut proteins. These were subsequently rapidly hydrolyzed by the enzyme, breaking the balance of the DPF aqueous solution system, and promoting protein dissolution into the aqueous phase. Therefore, the protein recovery was improved. Breaking the linkage between proteins and other constituents can also facilitate protein solubility [13].

Under the action of Flavorzyme, which contains a mixture of exopeptidases and endoproteases, peptide bonds in protein molecules were hydrolyzed more extensively through the joint action of endo- and exopeptidases, and the flavor of hydrolysates was better with more free amino acids. During the initial hydrolysis, the proteins were hydrolyzed into high-molecular-weight peptides by endoproteases, and then dissociated into smaller fragments by exoproteases [14]. As endonucleases, Alcalase and Protamex can assist Flavorzyme to improve the protein hydrolysis efficiency.

### 2.2. Taste Characteristics of DPH

**Figure 2:**
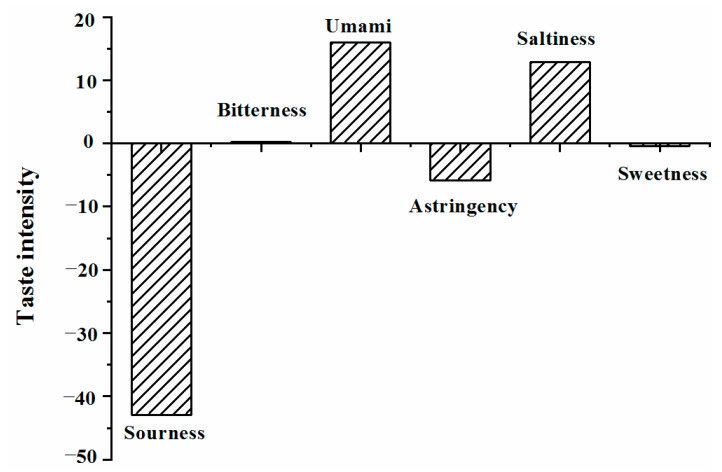
Enzymatic hydrolysis characteristics of DPF.

The sensory properties of proteins, especially the gustatory properties, can be increased by hydrolysis with certain proteases, such as Trypsin, Alcalase, and Flavorzyme [15,16]. An electronic tongue was used to objectively evaluate the taste characteristics of DPH, with the results shown in Figure 2. All data were absolute output values based on artificial saliva. The reference solution was regarded as a standard, and its taste intensity was calculated as 0 by the sensor response output. Using the test, the tasteless points of sourness, saltiness, and other tastes were found to be −13, −6, and 0, respectively, which were attributed to the reference solutions containing small amounts of acid and salt. When the taste value was less than the tasteless point value, no corresponding taste was present. Otherwise, a higher value corresponded to a stronger taste intensity.

DPH exhibited no sourness, sweetness, or astringency, and very slight bitterness, as the values were below the tasteless point value and around zero, respectively. However, umami was the major taste sense in the quantitative analysis, followed by saltiness, with values of 15.92 and 12.83, respectively. Regarding the function of taste interactions, Iwaniak et al. [17] confirmed that umami can enhance the salty taste of foods. On the other hand, sodium ions were introduced that adjusted the pH to make the DPH seem salty. These results suggested that DPH produces complex taste sensations and an outstanding umami taste. Therefore, DPH shows potential as a natural taste-based material.

### 2.3. Amino Acids Analysis

The total AA compositions and FAA contents in DPF and DPH are shown in Table 1. Both DPF and DPH were rich in Glu, Arg, and Asp but lacked Met, in agreement with previous studies [18,19]. No significant differences in the contents of most amino acid (AA) were observed between DPF and DPH (*p* > 0.05), indicating that the AA composition of DPF was not very affected by enzymatic hydrolysis. The umami AAs Asp and Glu accounted for total contents of about 34% by calculation. However, the contents of all AAs were decreased to some extent. Therefore, the protein might be partially enzymolyzed into FAA, with a small fraction of FAAs reacting with other compounds in DPF, such as sugars, or degrading during heating to produce volatile substances.

Owing to enzymolysis, the FAA content greatly increased from 0.86 to 23.59 g/100 g. FAAs have been reported to contribute directly to food taste [20,21]. Among the 17 types of FAA, Arg was dominant in DPH. Arg and its peptides are known to effectively amplify saltiness [22]. The content of umami FAAs increased substantially, with the Asp and Glu content being 28 and 9.05 times those in DPF, respectively. However, Asp and Glu accounted for a content of only 11.23% in DPH, which was lower than that of sweet FAAs (29.67%; Ser, Gly, Thr, Pro, Val, and Lys). Furthermore, the proportion of bitter FAAs (Arg, Leu, Phe, Tyr, Val, Ile, Lys, and Pro) reached 60.41%. However, according to the electronic tongue results, no sweet intensity and low bitterness were observed. This might be attributed to the presence of interactions between umami and other taste substances. Sweet substances appear to enhance the umami intensity [23]. The relationship between umami and bitterness, in terms of the taste receptor, was reported by Kim et al. [24,25], showing that umami substances suppress the bitterness of bitter amino acids or peptide solutions.

### 2.4. Molecular Weight Distribution of Peptides

**Figure 3:**
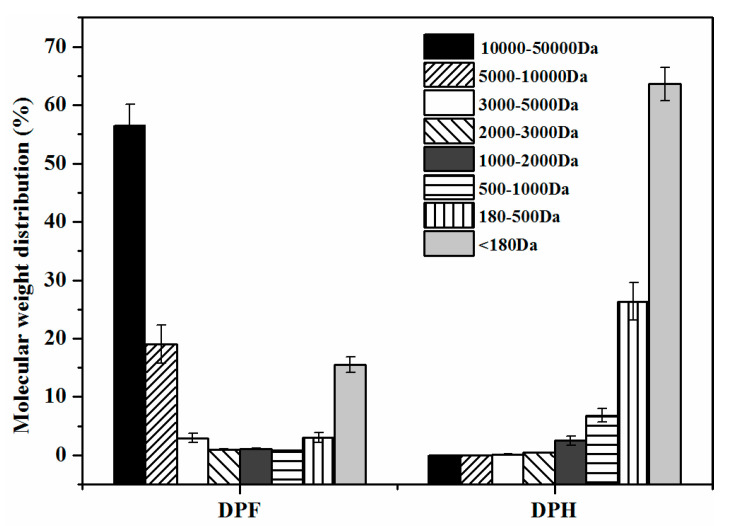
Molecular weight distribution of peptides.

The molecular weight distribution of peptides can also reflect the hydrolysis effect, with the results shown in Figure 3. The peptide in DPH could be divided into 8 parts according to the molecular weight (MW; >50, 10–50, 5–10, 3–5, 1–3, 0.5–1, 0.18–0.5, and <0.18 kDa). The molecular weight of peanut protein was mainly in the range of 10,000–50,000 Da (56.52%). DPF protein was clearly extensively hydrolyzed, and it was reasonable that many small molecular peptides were present in DPH compared with DPF. DPH contained large amounts of peptides and FAAs, with no protein components larger than 10,000 Da. Furthermore, the content of peptides with smaller molecular weights (<1000 Da) was up to 96.84%. These small molecular peptides were correlated with food flavor, and had a special effect on the taste characteristics. Apriyantono et al. [26] found that peptides of 500–1000 Da elicit a strong taste and had a positive taste effect. We speculated that the umami and umami-enhancing peptides were derived from these small molecular peptides.

### 2.5. FT-IR Spectroscopy

**Figure 4:**
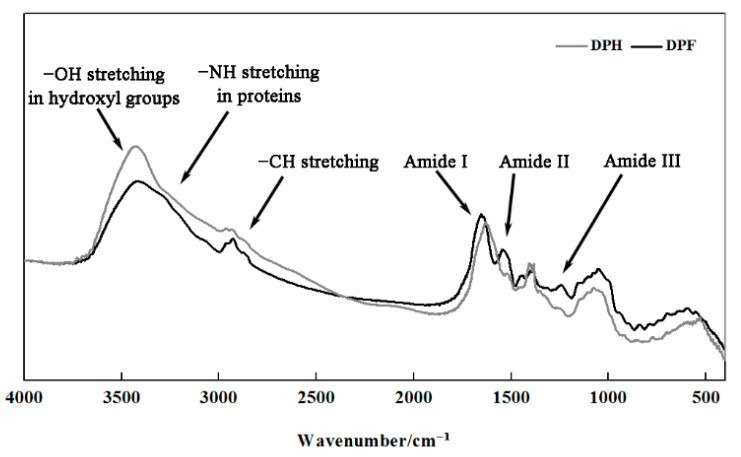
Fourier transform infrared (FT-IR) spectra of DPF and DPH.

Figure 4 shows the FT-IR absorbance spectra of DPF and DPH in the region of 4000–400 cm^−1^. Lipid was omitted in this research, because the lipid content of DPF determined by the Soxhlet method was less than 1%. For each characteristic absorption band, including 4000–3100 cm^−1^, 3100–2800 cm^−1^, and 3 amide bands [27,28,29], the absorbance spectroscopy of DPH was similar to that of DPF. However, differences were observed in the intensities and widths attributed to the formation of peptide bonds, and the protein content after hydrolysis [19].

The amide I band is deemed the most useful band for characterizing the secondary structure of proteins [30]. As shown in Figure 4, a redshift of the FT-IR maximum absorbance spectra was observed. The maximum absorbance was 1654 cm^−1^ for DPF but was shifted to 1630 cm^−1^ for DPH. This phenomenon might be caused by the aggregation of peptides during thermal treatment after hydrolysis.

The deconvolution method was used to fit the protein amide I band, obtaining the peak area of the protein secondary structures. The corresponding relationship between each characteristic peak and protein secondary structure was based on He et al. [31]. The results in Table 2 illustrate that the β-type conformation, including β-sheets and β-turns, was the major secondary structure in both DPF and DPH, but the secondary structure contents were different. Except for α-helixes, no significant differences were observed among the other structures (*p* > 0.05). With hydrolysis, the α-helixes decreased from 20.93% to 16.24% and β-sheets increased from 43.54% to 52.02%. α-helixes and β-sheets are relatively ordered structures [32]. When the protein structures were destroyed by enzymatic treatment, the change from one ordered structure to another occurred in the rearrangement process.

### 2.6. Ultrafiltration (UF) of DPH

To identify the key peptides that contributed to the intense taste, DPH was subjected to further purification. Ultrafiltration was used to partition DPH into 5 fractions, namely, UF-I (>10,000 Da), UF-II (5000–10,000 Da), UF-III (3000–5000 Da), UF-IV (1000–3000 Da), and UF-V (<1000 Da), which represented 5.01%, 9.87%, 14.72%, 21.54%, and 43.86% contents, respectively. According to the results of the peptide molecular weight distribution, DPH did not contain peptides of more than 10,000 Da but a very small amount of UF-II, which was inconsistent with the peptide molecular weight distribution results. As a possible explanation, UF is a membrane separation process relying on mechanical pressure, which makes the accurate separation of peptides with different molecular weights difficult [33]. Therefore, UF membranes in a small tangential flow UF system only had a preliminary separation effect.

Each fraction had a certain taste, but the results for bitterness (not tasted) were not in agreement with the electronic tongue measurements. This difference was likely due to the subjectivity of the assessors. The five fractions were qualitatively evaluated (Table 3). Depending on the electronic tongue results, umami and umami enhancement were evaluated. The umami intensity increased with decreasing molecular weight, with UF-V showing the highest umami intensity. Furthermore, these fractions were added at the 0.1% concentration to the 0.35% MSG–salt solution to evaluate the umami-enhancing effect. UF-V showed the best umami-enhancing effect (from 9.00 to 12.81) (*p* < 0.05), followed by UF-IV (increased to 9.50).

FAAs remained in each fraction, resulting from the inaccuracy of UF. Therefore, the other fractions also exhibited an umami taste. This result was in accordance with a previous report by Su et al. [12]. Furthermore, the interaction of peptides and FAAs, as demonstrated by Amin et al. [34], must be considered. UF-V possessed more small peptides, other than FAAs, as did UF-IV. These fractions exhibited evident umami and umami enhancement while other fractions did not.

### 2.7. Gel Filtration Chromatography Purification of Taste Peptides

**Figure 5:**
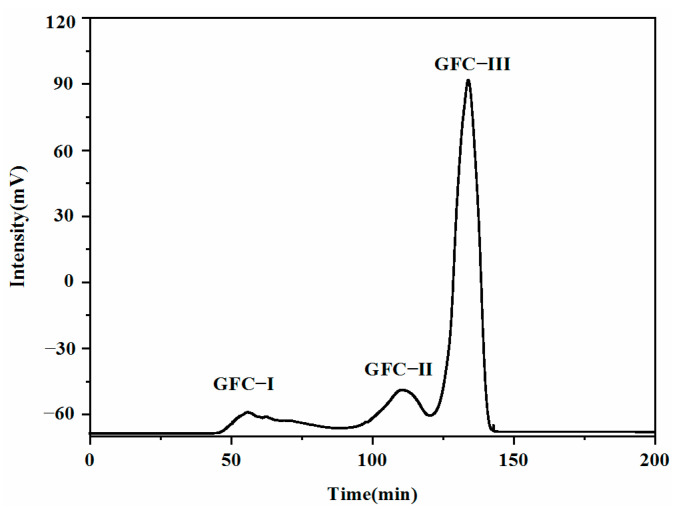
Gel filtration chromatography of the mixture of fractions GFC-I, GFC-II, and GFC-III.

Fraction UF-V, which had the strongest taste intensity, was purified by Sephadex G-15 filtration chromatography to obtain the strong umami taste components. The purification profile is shown in Figure 5. The fraction was purified into three components (GFC-I, GFC-II, and GFC-III), which were subjected to further analysis.

As shown in Table 3, GFC-III had an umami taste while GFC-I and GFC-II did not. GFC-II had a higher TD value for the enhancement effect (up to 14.57). Combined with the taste description results, GFC-III was identified as the component responsible for umami and selected for structural identification.

### 2.8. Taste Peptide Identification by UPLC-MS/MS

GFC-III was subjected to further analysis to identify the umami-enhancing peptides. Nineteen peptides all containing umami AAs were detected in GFC-III, with the basic information summarized in Table 4. The molecular weights of these peptides were all 500–1000 Da, which confirmed the aforementioned speculation. The peptide sequences were searched for on the BIOPEP database, but no corresponding umami and umami-enhancing peptides were found. Therefore, the acquired peptides were believed to be novel.

### 2.9. Sensory Evaluation of Synthetic Peptides

To verify the taste features of the identified peptides, they were synthesized and subjected to sensory evaluation (Table 5). All synthetic peptides had no umami taste. Some exhibited sourness and astringency while the others were tasteless. The astringent taste might be derived from the presence of acetate residues during the synthesis process [35]. The sourness might be caused by residual AAs during target peptide synthesis [8]. The umami enhancement effect was tested. Among the synthetic peptides, five umami-enhancing peptides were discovered, namely, ADSYRLP, DPLKY, EAFRVL, EFHNR, and SDLYVR. These peptides possessed different threshold values that exhibited different synergistic effects with MSG. In the presence of MSG, ADSYRLP had the lowest threshold value, which suggested the largest umami enhancement effect on MSG (*p* < 0.05).

In this study, single synthetic peptides had no umami taste, but their mixtures obtained by GCP did, probably resulting from the spatial structure [36]. The synthetic peptides probably had various conformations different from those in the natural state, preventing them from properly binding to the umami taste receptors. Many reports have proven the presence of umami enhancement, with much attention paid to the mechanism. Escriche et al. [37] confirmed that synergistic umami might be attributed to the spatial conformation of umami receptor proteins. Later, Yoshida et al. [38] found that the mechanism of synergistic umami was allosteric regulation. Therefore, this synergy was due to MSG binding with the receptor protein, eliciting an altered spatial conformation, and then peptides bound with the receptor protein.

## 3. Materials and Methods

### 3.1. Materials

DPF (protein content, 56%) passed through an 80-mesh sieve was obtained from the National Center for Peanut Improvement, Henan Peanut Subcenter (Zhengzhou, China). Alcalase 2.4 L FG, Protamex, and Flavorzyme 500 MG were provided by Novozymes Biotechnology Co., Ltd. (Tianjin, China). The multiprotease was composed of Flavorzyme 500 MG (4000 U/g), Alcalase 2.4 L FG (500 U/g), and Protamex (600 U/g). *O*-Phthaldialdehyde (OPA) was purchased from Aladdin Industrial Corporation (Shanghai, China). MSG was purchased from Meihua Biotechnology Group Co. Ltd. (Lhasa, Xinjiang, China). HPLC was performed using HPLC-grade chemicals and reagents purchased from Sigma-Aldrich Chemical Co. (St. Louis, MO, USA). All other chemicals and reagents were commercially available and of analytical grade.

### 3.2. Preparation of DPF Hydrolysate (DPH)

DPF was dispersed in deionized water (1:13, *w*/*w*) and pretreated for 30 min at 120 °C in an autoclave (Shanghai Shenan Instrument Co., Ltd., Shanghai, China). The resulting DPF dispersion was hydrolyzed with the multiprotease (5100 U/g) at pH 8.3 and 47 °C for 14 h in a water bath. The resulting hydrolysate was heated for 10 min in a boiling water bath to inactivate the enzyme, followed by centrifugation at 5000× *g* for 20 min using a CR22G high-speed centrifuge (Hitachi Co., Tokyo, Japan). The collected supernatant was lyophilized and stored at −18 °C prior to use.

### 3.3. Determination of Enzymatic Properties

#### 3.3.1. Protein Recovery

The protein recovery was calculated as the ratio of the protein amount present in the hydrolysates to that in DPF. The protein content was determined using the Kjeldahl method with a nitrogen conversion factor of 5.46.

#### 3.3.2. Degree of Hydrolysis (DH)

DH was determined using an OPA method [39]. The samples were appropriately diluted according to the protein content and expected DH. OPA (180 μL) was mixed with the test sample (24 μL), water (control), and 0.97 mM serine (standard). The absorbances of the OPA solution were measured at 340 nm after 2 min using a microplate reader (Infinite 200 PRO; TECAN (Shanghai) Trading Co., Ltd., Shanghai, China).

#### 3.3.3. The Yield of Peptide

The peptide yield was determined using the trichloroacetic acid (TCA) and biuret method [40]. The results were expressed as the ratio of peptide content in the enzymolysis solution to the protein content in DPF.

### 3.4. Molecular Weight (MW) Distribution of Peptides

The MW distribution of peptides in the supernatants from DPF and DPH were determined by gel permeation chromatography (P230, Elite Analytical Instrument Co., Ltd., Dalian, China) using an SEC-125 column (XIYU Tech, Shanghai, China). The mobile phase was acetonitrile/water/trifluoroacetic acid (45:55:0.1, *v*/*v*/*v*) at a flow rate of 0.5 mL/min. The detection wavelength was 220 nm.

### 3.5. Amino Acids Analysis

Amino acids (AAs) and free amino acids (FAAs) were analyzed. The pretreatment method was in accordance with the method of Wang et al. [41]. The supernatant obtained by pretreatment was mixed with the same volume of an amino acid standard working solution, and then injected into the amino acid analyzer (Model L-8900; Hitachi High Technologies, Dallas, TX, USA). Sodium citrate buffer and ninhydrin solution were used as the mobile phase and derivatization agent, respectively. The concentration of amino acids in the samples was calculated from the peak area obtained by the external standard method.

### 3.6. FT-IR Spectroscopy

After mixing DPH or DPF with potassium bromide in a mass ratio of 1:200, the mixture was fully ground with an agate mortar and pressed into transparent slices with a 15-ton hydraulic press. The FT-IR absorbance spectra of the samples were acquired using a Nicolet iS5 spectrometer (Thermo Fisher, Waltham, MA, USA) with a Golden Diamond Horizontal Attenuated Total Reflectance sampling device (Specac Ltd., Orpington, UK). Spectra (128 accumulated scans at aa 2 cm^−1^ resolution) were collected over the frequency range of 4000–400 cm^−1^. A sample background was collected in air before sample analysis.

### 3.7. Isolation and Purification of Peptides

#### 3.7.1. Ultrafiltration (UF)

The lyophilized DPH was dissolved in deionized water and fractionated using a mini ultrafiltration system (Mini Pellicon, Millipore Corporation, Burlington, MA, USA) through ultrafiltration membranes with selected molecular weight cut-offs of 10, 5, 3, and 1 kDa, respectively. UF-I, UF-II, UF-III, UF-IV, and UF-V represent fractions with molecular weight distributions of >10, 5–10, 3–5, 1–3, and <1 kDa, respectively. All fractions recovered were lyophilized for sensory evaluation.

#### 3.7.2. Gel Filtration Chromatography

The lyophilized fractions below 3 kDa were redissolved in ultrapure water to obtain a nitrogen concentration of 100 mg/mL, loaded onto a Sephadex G-15 gel filtration column (26 mm × 600 mm) at 25 °C, and eluted with ultrapure water at a flow rate of 1.25 mL/min. The UV absorbance of the effluent was monitored at 280 nm using a STI UV 501 spectrophotometer (Science Technology Co., Hangzhou, China) owing to the peptide bond absorbance at 280 nm, which yielded the greatest sensitivity. Fractions with the desired peptide peaks were pooled, concentrated, and lyophilized for sensory evaluation.

### 3.8. Identification of Peptides by UPLC-MS/MS

After gel filtration chromatography separation, the dried fractions with the most intense umami taste or umami-enhancing effect were reduced by 10 mM dithiothreitol at 56 °C for 1 h, alkylated by 50 mM iodoacetamide at 25 °C in the dark for 40 min, and lyophilized to near-dryness. The dried derivative samples were dispersed in 0.1% formic acid (2–20 μL) before UPLC-MS/MS analysis.

UPLC-MS/MS was conducted using a Q Exactive Hybrid Quadrupole-Orbitrap mass spectrometer (Thermo Fisher Scientific, San Jose, CA, USA) with an Ultimate 3000 system and a reversed-phase Repro Sil-Pur C18-AQ resin column (150 μm × 15 cm, 1.9 μm, Dr. Maisch GmbH, Ammerbuch, Germany). The 2 mobile phases were 0.1% (*v*/*v*) formic acid in water (A) and 0.1% formic acid in acetonitrile (B), at a flow rate of 600 nL/min. The gradient elution conditions were as follows: 4–8% B over 2 min, 8–28% B over 43 min, 28–40% B over 10 min, 40–95% B over 1 min, and 95% B for 10 min. The injection amount was 5.0 μL and the column temperature was 25 °C. Spectra were obtained in positive reflector mode at a spray voltage of 2.2 kV in the m/z range of 300–1800 with collision-induced dissociation (CID).

### 3.9. Peptide Synthesis

To determine the taste features of peptides identified by UPLC-MS/MS, they were synthesized by Beijing Bio-Tech Pack Technology Co., Ltd. (Beijing, China) using the solid-phase method. The peptide purity was >95%. Sensory profiling was used to verify the taste characteristics of the synthesized peptides.

### 3.10. Taste Traits Analysis Using an Electronic Tongue

The taste traits of DPH were analyzed according to Dang et al. [42] using a potentiometric method with an electronic tongue system (TS-5000Z, INSENT, Tokyo, Japan). A 20 mg/mL DPH solution was prepared with ultrapure water and placed in the system for 30 min. The reference solution contained 30 mM KCl and 0.3 mM tartaric acid.

### 3.11. Sensory Profiling

Sensory analysis was conducted by 10 panelists (5 men and 5 women, aged 25–40) trained in similar sensory experiments for at least 1 year. The panelists gave informed consent to participate in sensory tests in the present investigation. Tasting sessions were conducted in an independent room with an individual table at 25 ± 2 °C.

Taste dilution (TD) analysis [43,44] was performed to determine the umami threshold of peptides and TD values of DPH and fractions from UF and GFC. Each sample with or without MSG–salt solution was diluted gradually using deionized water until the panelists could not identify the sample solution from two parts of deionized water [45]. The threshold value was the molar concentration of the peptide in the solution at the end point of dilution. The TD value was the amount of deionized water added at the end point of dilution, expressed in mL. The 0.1% concentration samples were dissolved in 10 g of MSG (0.35%, w)–salt solution (0.35%, w), which was used to evaluate the umami-enhancing effect. The solution without MSG–salt was used to evaluate the umami taste intensity. Each sensory panelist was also asked to describe the taste of each sample.

### 3.12. Statistical Analysis

All data were subjected to statistical analysis using IBM SPSS Statistics 20.0 software (SPSS Inc., Chicago, NY, USA). Differences in the mean values of the data were evaluated using one-way analysis of variance (ANOVA) and Duncan’s multiple range test. Values of *p* < 0.05 were considered statistically significant. Figures were drawn using Origin 8.5 software (Origin Lab., Northampton, MA, USA).

## 4. Conclusions

This study showed that peanut protein hydrolysate has an umami taste and umami-enhancing effect. Free amino acids and low-molecular-weight peptides (<3000 Da) made important contributions to the umami taste of peanut enzymatic hydrolysates. Five umami-enhancing peptides from peanut hydrolysate were purified and identified. These novel peptides with the sequences ADSYRLP, DPLKY, EAFRVL, EFHNR, and SDLYVR were recognized to enhance the umami taste intensity of an MSG–salt solution. Owing to the diversity of peptides and complex nature of umami taste, the mechanism through which the peptide structure affects taste remains unclear, further work is needed to explore for the directional guidance of umami peptide production.

## Figures and Tables

**Table 1:** AA compositions and FAA contents.

Species	AAs (g/100 g)		FAAs (g/100 g)	
	DPH	DPF	DPH	DPF
Asp	5.92 ± 0.40 ^b^	7.03 ± 0.16 ^a^	0.84 ± 0.00 ^a^	0.03 ± 0.00 ^b^
Glu	10.42 ± 0.35 ^b^	12.51 ± 0.28 ^a^	1.81 ± 0.04 ^a^	0.20 ± 0.00 ^b^
Ser	2.26 ± 0.35 ^a^	2.70 ± 0.23 ^a^	2.27 ± 0.11 ^a^	0.03 ± 0.00 ^b^
Arg	5.97 ± 0.23 ^b^	7.28 ± 0.33 ^a^	3.77 ± 0.17 ^a^	0.07 ± 0.00 ^b^
Gly	2.73 ± 0.21 ^a^	3.32 ± 0.25 ^a^	0.70 ± 0.00 ^a^	0.01 ± 0.00 ^b^
Thr	1.34 ± 0.06 ^a^	1.57 ± 0.02 ^a^	1.00 ± 0.00 ^a^	0.01 ± 0.00 ^b^
Pro	2.05 ± 0.41 ^a^	2.49 ± 0.17 ^a^	0.32 ± 0.00 ^a^	0.17 ± 0.00 ^b^
Ala	1.97 ± 0.14 ^a^	2.37 ± 0.24 ^a^	1.14 ± 0.01 ^a^	0.03 ± 0.00 ^b^
Val	2.17 ± 0.10 ^a^	2.53 ± 0.23 ^a^	1.63 ± 0.04 ^a^	0.04 ± 0.00 ^b^
Met	0.44 ± 0.00 ^a^	0.49 ± 0.00 ^a^	0.32 ± 0.00 ^a^	0.01 ± 0.00 ^b^
Ile	1.72 ± 0.14 ^a^	2.03 ± 0.08 ^a^	1.34 ± 0.06 ^a^	0.01 ± 0.00 ^b^
Leu	3.17 ± 0.13 ^b^	3.92 ± 0.28 ^a^	2.47 ± 0.14 ^a^	0.01 ± 0.00 ^b^
Phe	2.81 ± 0.10 ^a^	3.47 ± 0.42 ^a^	1.89 ± 0.14 ^a^	0.03 ± 0.00 ^b^
His	1.13 ± 0.00 ^a^	1.39 ± 0.19 ^a^	0.69 ± 0.02 ^a^	0.01 ± 0.00 ^b^
Lys	1.84 ± 0.08 ^a^	2.27 ± 0.25 ^a^	1.08 ± 0.00 ^a^	0.02 ± 0.00 ^b^
Tyr	1.95 ± 0.07 ^a^	2.12 ± 0.30 ^a^	1.75 ± 0.00 ^a^	0.15 ± 0.00 ^b^
Total	47.89 ± 0.08 ^b^	57.51 ± 0.83 ^a^	23.30 ± 0.13 ^a^	0.85 ± 0.00 ^b^

Different superscript letters within the same row denote significant differences (*p* < 0.05).

**Table 2:** Protein secondary structures of DPF and DPH.

	α-Helix	β-Sheet	β-Turn	Random Coil
DPF	20.93 ± 2.00 ^a^	43.54 ± 2.56 ^b^	24.40 ± 1.62 ^a^	11.14 ± 1.53 ^a^
DPH	16.24 ± 1.55 ^b^	52.02 ± 3.29 ^a^	23.61 ± 2.50 ^a^	8.13 ± 0.76 ^a^

Different superscript letters within the same column denote significant differences (*p* < 0.05).

**Table 3:** Sensory evaluation of UF and GFC fractions.

Fraction	Without MSG-Salt		With MSG-Salt	
	Taste Description	TD Value	Umami Enhancement	TD Value
MSG-salt	--	--	Umami	7.50 ± 0.36
DPH	Strong umami, salty	3.25 ± 0.29	Strong umami	9.00 ± 0.49
UF-I	Slight umami	0.90 ± 0.00	Not detected	--
UF-II	Slight umami	1.23 ± 0.02	Not detected	--
UF-III	Umami	1.36 ± 0.02	Not detected	--
UF-IV	Umami	2.22 ± 0.17	Strong Umami	9.50 ± 0.66
UF-V	Strong umami	4.87 ± 0.20	Strong umami	12.81 ± 0.54
GFC-I	Tasteless	--	Not detected	--
GFC-II	Tasteless	--	Not detected	--
GFC-III	Strong umami	5.44 ± 0.34	Strong Umami	14.57 ± 0.82

TD—taste dilution.

**Table 4:** Obtained peptide information.

Peptide	Scan	Score	Length	m/z	z	Mass
ADSYRLP	6995	99	7	411.2096	2	820.4079
DAQRPF	5396	97	6	367.1835	2	732.3555
DFRAP	5639	99	5	303.1544	2	604.2969
DPLKY	4887	99	5	318.1722	2	634.3326
DQFPR	4274	99	5	331.6651	2	661.3184
DSRPF	4986	98	5	311.1518	2	620.2918
DWRQERP	4321	91	7	329.4971	3	985.473
EAFRVL	8208	99	6	367.7119	2	733.4122
EFHNR	1617	99	5	351.6681	2	701.3245
EWAGLTTN	8189	90	8	446.2134	2	890.4134
KDNNPF	5065	94	6	367.675	2	733.3395
LDAQRP	2816	99	6	350.1914	2	698.3711
LDQFPR	5904	99	6	388.207	2	774.4024
LDQFRP	5497	97	6	388.2063	2	774.4024
NDFGR	2762	98	5	304.6415	2	607.2714
NDNPFKF	10,478	95	7	441.2111	2	880.4079
SDLYVR	4539	99	6	376.6993	2	751.3864
VPPFDHQ	6019	95	7	420.204	2	838.3973
VPQDFR	4362	99	6	381.1999	2	760.3868

Peptide is the amino acid sequence; scan is the mass spectrum number; score was obtained by de novo calculation; length is the number of amino acids; m/z is the peptide mass/charge ratio; z is the peptide charge; mass is the peptide molecular weight.

**Table 5:** Descriptive sensory evaluation of synthetic peptides and their MSG-enhanced solutions.

Synthetic Peptides	Without MSG-Salt		With MSG-Salt	
	Taste Description	Threshold Value (mmol/L)	Umami Enhancement	Threshold Value (mmol/L)
ADSYRLP	Sour	--	Strong umami	0.107 ± 0.004 ^a^
DAQRPF	Tasteless	--	Not detected	--
DFRAP	Tasteless	--	Not detected	--
DPLKY	Sour	--	Slight umami	0.164 ± 0.008 ^d^
DQFPR	Tasteless	--	Not detected	--
DSRPF	Tasteless	--	Not detected	--
DWRQERP	Tasteless	--	Not detected	--
EAFRVL	Sour, astringent	--	Umami	0.134 ± 0.004 ^bc^
EFHNR	Sour, astringent	--	Umami	0.148 ± 0.004 ^c^
EWAGLTTN	Sour	--	Not detected	--
KDNNPF	Tasteless	--	Not detected	--
LDAQRP	Sour, astringent	--	Not detected	--
LDQFPR	Tasteless	--	Not detected	--
LDQFRP	Sour	--	Not detected	--
NDFGR	Tasteless	--	Not detected	--
NDNPFKF	Sour, astringent	--	Not detected	--
SDLYVR	Tasteless	--	Strong umami	0.132 ± 0.007 ^b^
VPPFDHQ	Tasteless	--	Not detected	--
VPQDFR	Astringent	--	Not detected	--

Different superscript letters within the same column denote significant differences (*p* < 0.05).

## Data Availability

Not applicable.

## Sample Availability

Samples of the compounds are not available from the authors.
